# Hybrid polymer photonic crystal fiber with integrated chalcogenide glass nanofilms

**DOI:** 10.1038/srep06057

**Published:** 2014-08-14

**Authors:** Christos Markos, Irnis Kubat, Ole Bang

**Affiliations:** 1 DTU Fotonik, Department of Photonics Engineering, Technical University of Denmark, DK-2800 Kgs. Lyngby, Denmark

## Abstract

The combination of chalcogenide glasses with polymer photonic crystal fibers (PCFs) is a difficult and challenging task due to their different thermo-mechanical material properties. Here we report the first experimental realization of a hybrid polymer-chalcogenide PCF with integrated As_2_S_3_ glass nanofilms at the inner surface of the air-channels of a poly-methyl-methacrylate (PMMA) PCF. The integrated high refractive index glass films introduce distinct antiresonant transmission bands in the 480–900 nm wavelength region. We demonstrate that the ultra-high Kerr nonlinearity of the chalcogenide glass makes the polymer PCF nonlinear and provides a possibility to shift the transmission band edges as much as 17 nm by changing the intensity. The proposed fabrication technique constitutes a new highway towards all-fiber nonlinear tunable devices based on polymer PCFs, which at the moment is not possible with any other fabrication method.

The photonic crystal fiber (PCF) is an optical fiber in which the cladding is formed by a periodic array of micron sized holes running along the entire length of the fiber^1^. The hole-structure defines the fiber properties and consequently PCFs have attracted a lot of scientific attention, since they offer unique design flexibility and provide an amazing degree of freedom for manipulation of their guiding properties. The PCF can for example be made single-mode at all wavelengths^1^, the zero-dispersion wavelength (ZDW) may be moved relatively easy, even down to the visible^2^, and they can be made to guide light in air^3^. These properties are not possible in standard optical fibers.

The PCF is mainly fabricated from a single material, which can be glass^1^,^2^,^3^,^4^,^5^, polymer^6^,^7^,^8^,^9^ or even a combination of different materials^10^,^11^,^12^, depending on the desired application. For example, silica PCFs are widely used in wavelength conversion and supercontinuum sources^13^ due to their low material loss and tunable zero-dispersion, while on the other hand polymer PCFs have been used mainly for sensing applications, such as strain sensing^14^,^15^,^16^,^17^ and fiber-optical biosensing^18^,^19^, due to their low Young's modulus and biocompatibility properties, respectively. Another distinct advantage of PCFs is their unique ability to host novel and functional materials in their cladding holes providing the possibility for completely different guiding mechanisms^20^,^21^. It has been demonstrated already that the guidance mechanism of a silica PCF can be converted from index- to bandgap or anti-resonant reflecting optical waveguide (ARROW)^20^ guidance by incorporating liquid crystal^22^, high index liquids/polymers^23^, glasses^24^,^25^, high index films^26^,^27^,^28^, etc. as active materials inside the holes of the fiber. These infused functional materials can be highly tunable using different external perturbations, such as temperature or an applied electric field, thereby affecting the guiding properties of the fiber and allowing the development of all-fiber devices and sensors^29^. However, so far only limited to no research has been carried out on the combination of polymer PCFs with active nonlinear glasses for the development of all-fiber nonlinear devices.

Chalcogenide glasses are considered ideal optical materials for the development of all-fiber nonlinear devices, since they exhibit an extremely high Kerr nonlinear coefficient, can transmit light in the mid-IR, and have low two photon absorption and a fast response time^30^, in contrast to for example polymer materials. Chalcogenide glasses have another important property, which is their photosensitivity when exposed to light near their bandgap edge, which further support their candidacy for all-optical tunable devices^30^. In this article, we experimentally demonstrate how to combine the stoichiometric As_2_S_3_ chalcogenide glass with a PMMA-based PCF by using a simple and cost-effective technique, based on nanocolloidal solution-based glass^31^. Previous reports have demonstrated the melt-filling pressure-assisted technique in silica PCFs, where either tellurite or chalcogenide glass is molten at high temperature (i.e. 600–800°C) and high pressure is then applied to infiltrate the molten glass into the holes of the silica PCF^24^,^32^. However, this technique requires expensive, custom-made and sophisticated equipment and cannot be used for polymer PCFs, since the melting point of PMMA is at much lower temperatures (~120°C) than for tellurite or chalcogenide. Furthermore, the required pressure will easily destroy the air-hole pattern of the cross-section of the polymer PCF.

After the preparation of the nanocolloidal glass solution, the material was infiltrated into the holes of the PCF at room temperature. By annealing the fiber at low temperature (~65°C), the solvent was then evaporated, forming consequently a nanometer-thick layer of the desired chalcogenide glass at the inner surface of the holes in the polymer PCF^28^. Importantly, the thickness of the final formed films can be controlled by modifying the concentration of the nanocolloidal solution-based glass. Furthermore, the thickness is depending on the annealing temperature of the fiber and the evaporation rate of the solvent. The proposed solution-based method is presently the only way to combine the polymer PCF with nonlinear glasses. Furthermore, the proposed simple technique enables an enormous flexibility in the choice of what chalcogenide glass one wants to use and provides a possibility for multi-layer deposition of different glass materials inside the holes of a polymer PCF or even in planar waveguide structures for mid-IR applications^33^.

## Results

### Hybrid PCF characterization

**Figure 1:**
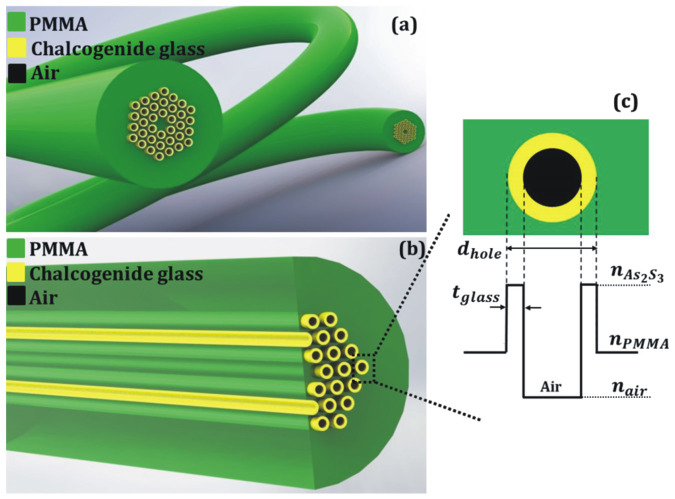
(a) Schematic representation of the polymer PCF with chalcogenide glass nanofilms. (b) Side zoomed view of the proposed fiber structure. (c) Refractive index profile of a single hole.

A schematic representation of the proposed hybrid polymer PCF with the integrated chalcogenide glass films is shown in Fig. 1(a). Figure 1(b) shows a side zoomed view of the structure across the length of the fiber and 1(c) the refractive index profile of a single hole of the fiber where *d_hole_* and *t_glass_* corresponds to the initial diameter of the cladding hole and the thickness of the formed chalcogenide glass film, respectively.

**Figure 2:**
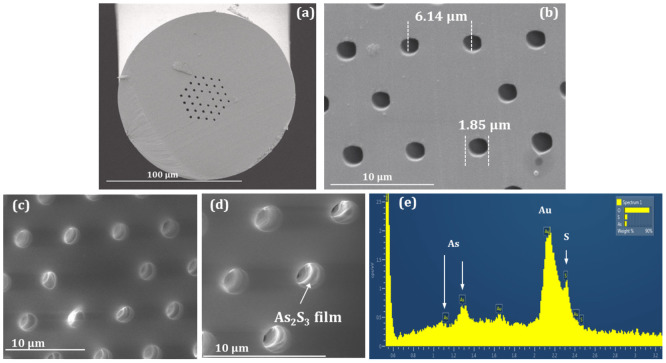
(a) SEM image of the fabricated PMMA PCF. (b) Magnified SEM image with the structural dimensions. (c) SEM image of the hybrid chalcogenide glass/polymer PCF showing the deposition of glass films in the holes using the nanocolloidal solution-based chalcogenide glass with concentration of ~50 mg/mL. (d) Magnified area of the cross-section showing the chalcogenide film more clearly. (e) Energy dispersive X-ray spectroscopy verifying the existence of the Arsenic and Sulfide elements on the inner wall of the air-holes.

The polymer PCF used in our investigations is made in-house using the drill-and-draw technique (see Methods). Figure 2(a) shows a Scanning Electron Microscopy (SEM) image of the fabricated polymer PCF with an outer diameter of ~125 μm. Figure 1(b) shows a magnified SEM image of the fiber with the structural dimensions. The endlessly single-mode condition for a PCF is defined as when the ratio of the hole diameter *d* to the pitch *Λ* (distance between the holes) is *d*/*Λ* < 0.42^34^. Our fabricated polymer PCF has *d*/*Λ* ~ 0.34 ensuring thus that the fiber supports only the fundamental mode at all wavelengths^1^. In order to investigate the role of the concentration to the final thickness of the glass layers inside the holes of the fiber, two different chalcogenide glass solutions were prepared with concentration ~50 mg/mL and ~400 mg/mL (see Methods). A short piece of the polymer PCF was first immersed into the solution with concentration ~50 mg/mL. The capillary forces enabled the infiltration of the chalcogenide glass solutions over ~4 cm in length in a few minutes, due to the low viscosity of the material^35^. The hybrid PCF was rested at room temperature for 24 h and was then annealed at ~65°C for 5 h in order to evaporate the solvent and leave only the glass layer. The SEM image in Fig. 2(c) shows the deposition of the nanometer-scaled chalcogenide glass layer inside the holes of the polymer PCF, with the zoom in Fig. 2 (d) more clearly showing the presence of the chalcogenide glass layers (see Supplementary Information). In order to further confirm the deposition of the glass films on the surface of the holes, we used Energy dispersive X-ray spectroscopy (EDX) for an element analysis (see Supplementary Information). Figure 1(e) shows the EDX spectrum of a small area of the cross-section of the fiber (Fig. 1(d)), which verifies the existence of the As (arsenic) and S (sulfide) lines. The Au (gold) peaks in the same spectrum arise from the gold deposited on the end-facet of the fiber for the SEM characterization. The thickness of the chalcogenide films in this case could not be accurately determined, since it was only a few nanometers thick.

**Figure 3:**
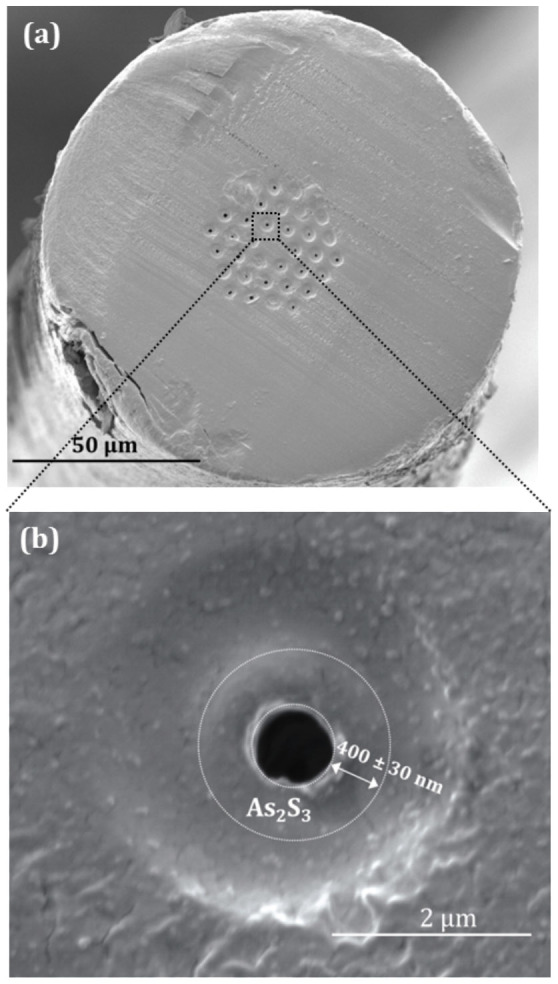
SEM images of the hybrid chalcogenide/PMMA PCF fabricated with a concentration of ~400 mg/mL. (a) Entire cross-section of the fiber. (b) Magnified single hole with chalcogenide film of thickness 400 nm ± 30 nm. The initial diameter of the hole was *d* = 1.85 *μm*.

Using the same conditions, a new hybrid polymer PCF sample was prepared using the highest concentration (~400 mg/mL), in which case a much thicker film was deposited on the inner surface of the holes. Figure 3(a) shows a SEM image of the new fiber sample, which clearly shows the deposition of the chalcogenide glass films in the holes. The uniformity of the glass films across the end facet of the fiber cannot adequately be determined due to the distorted cross section of the fiber introduced after the cleaving of the fiber^36^. Figure 3(b) shows the magnification of a single hole, from which the thickness of the As_2_S_3_ layer is estimated to be ~400 nm. In order to confirm the existence and the consistency of the formed glass nanofilms along the length of the fiber, the fiber was cleaved after ~2 cm. An investigation of the end facet verified the relative consistency of the films (see supplementary Figs. S1 and S2).

### Simulations and optical measurements

In general, high refractive index inclusions in the cladding holes of a PCF can modify the waveguiding mechanism and introduce minima and maxima in the transmission spectrum^20^. The thickness of the deposited high index glass films in the cladding holes of the PCF has a crucial role for the guiding properties. When the deposited chalcogenide glass films for example are so thin that they cannot support guided modes, then the guided core mode can couple to Mie-like resonances introducing dips in the transmission. These dips occur at wavelengths where the dispersion curves for the high index-resonances and polymer PCF core-mode anti-cross, causing light to couple strongly to the As_2_S_3_ and enhancing the loss^37^. On the other hand, when the high index chalcogenide films are thick enough to support modes, the minima in the transmission can be predicted using the ARROW model^20^,^38^. In the case where the holes of the fiber are completed homogeneously filled with the chalcogenide glass (i.e. forming solid rods), then the light guiding mechanism can be described using either the ARROW or bandgap guidance theory^3^. Modifying the composition of the material, the semiconducting nature of amorphous chalcogenide glass could possibly introduce an extra guiding mechanism based on the excitation of plasmonic modes at the high-index glass layers. Plasmonic effects in metal filled PCFs have been previously demonstrated^39^. Therefore, the hybrid polymer PCF could offer a large flexibility in designing all-fiber devices with a desired guiding mechanism.

**Figure 4:**
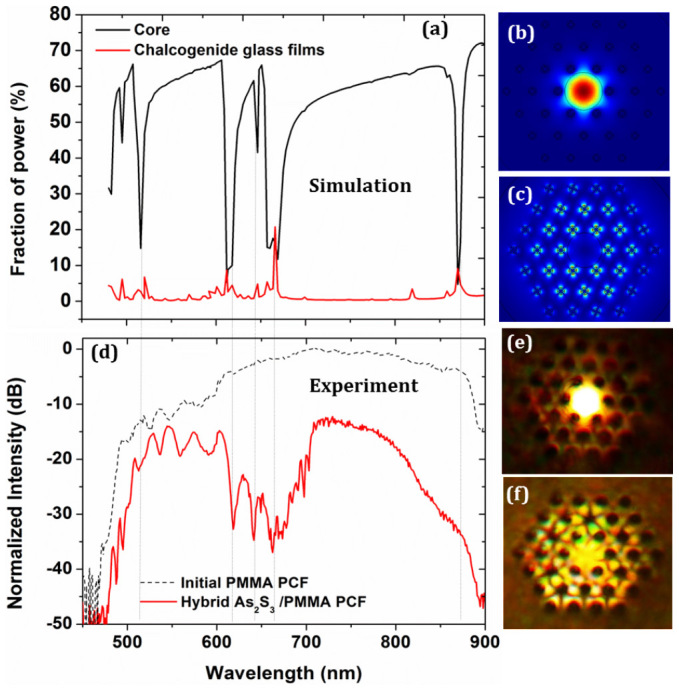
(a) Calculated fraction of power in the core of the hybrid chalcogenide/PMMA PCF (black line) and fraction of power in the high-index chalcogenide glass films (red line) for chalcogenide glass films of thickness 400 nm. (b) Simulated near-field profile of the fundamental guided mode at 750 nm where the transmission is high. (c) Simulated profile at 870 nm where the transmission is minimum, indicating coupling of the light to the high-index As_2_S_3_ layers. (d) Experimental measurement of the transmission spectrum of the initially fabricated polymer PCF (black dotted line) and the hybrid As_2_S_3_/PMMA PCF (red line). The transmission minima of the experimental transmission spectrum are in relatively good agreement with the predicted spectrum for a of thickness 400 nm as indicated by the blue dotted lines. (e) Near-field profile of the excited fundamental mode. (f) Near-field profile of the excited modes in the high-index chalcogenide glass films.

Here we use the numerical Finite Element Method (FEM) to simulate the guiding properties of the hybrid polymer PCF (see Supplementary Information), considering the As_2_S_3_ film thickness to be 400 nm and predict the frequencies where the transmission spectrum exhibits minima. Figure 4 (a) presents the fraction of power (%) in the core of the fundamental guided mode of the hybrid As_2_S_3_ polymer PCF (black line) in the 450–900 nm wavelength region. There are 4 distinct windows with a high fraction of power in the core, defined as transmission windows, with bandwidths 100 nm (515–615 nm), 30 nm (615–645 nm), 15 nm (645–660 nm) and 210 nm (660–870 nm). At the transmission minima of the fundamental mode, there is a corresponding increase in the fraction of power in the high-index chalcogenide films (red line). Figures 4(b) and 4(c) show the calculated mode profile of the fundamental mode at 750 nm and 870 nm, respectively. When the transmission is maximum (off-resonance state in ARROW terminology), the light is confined in the core of the fiber as shown at Fig. 4(b). Similarly, when the transmission exhibits a minimum (on-resonance state), the fundamental mode is coupled to the high-index chalcogenide glass films, as shown in Fig. 4(c).

With the numerical results in mind we experimentally characterized the hybrid polymer PCF using a high power supercontinuum laser source with a spectrum covering 480–2200 nm (see Methods). Figure 4(d) shows the transmission spectrum of the initially fabricated index-guiding polymer PCF of length ~15 cm (black dotted line). Repeating the measurements with the new hybrid polymer PCF of length L ≈ 4 cm, a clear signature of ARROW guidance is observed, as shown in Fig. 4(d) (red line). The new hybrid polymer PCF exhibits transmission windows similar to the ones predicted by numerical simulation with a relatively good agreement between the calculated and measured minima (black dotted lines connecting Figs. 4(a) and 4(d)). The short wavelength limit of the supercontinuum source is 480 nm, which is why there is no transmission below 480 nm. It should also be noticed that non-uniformities of the chalcogenide glass films along the length of the fiber, combined with small variations of the refractive index of the formed chalcogenide glass could be a possible source for variations in the intensity and the precise location of the transmission bands. The oscillations appearing between 500–600 nm at Fig. 4(d) are dependent also on the launching conditions. Figure 4(e) shows the captured white-light near-field profile of the fundamental guided mode of the hybrid As_2_S_3_/PMMA PCF when the light from the supercontinuum source is coupled to the core of the fiber. By slightly modifying the launch conditions, the light can be easily coupled to the high-index glass films and excite their resonant modes, as it can be seen from the near-field image shown in Fig. 4(f), which further confirms the existence of the high-index glass films in the holes of the fiber.

**Figure 5:**
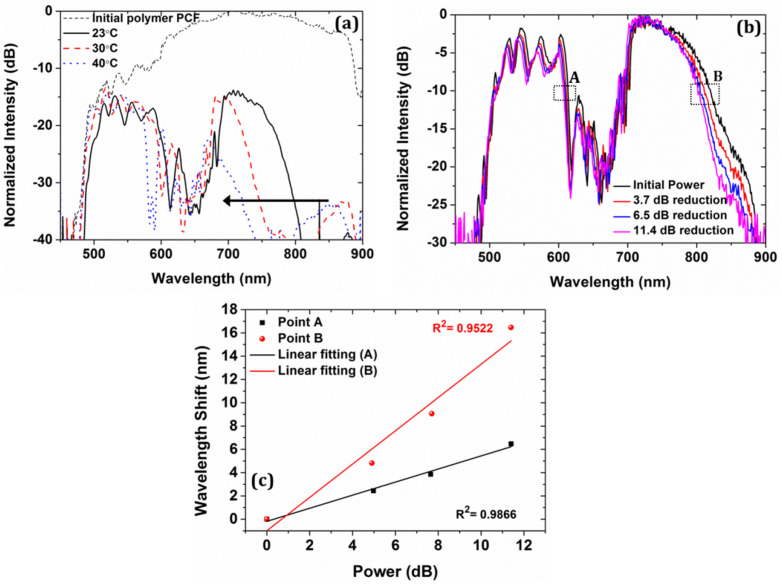
Optical characterization of the hybrid As_2_S_3_/PMMA PCF. (a) Thermal response of the transmission bands at 23°C, 30°C and 40°C. (b) Normalized transmission of the hybrid fiber and shift of the transmission edge at different power levels. (c) Linear behavior of the Kerr effect of the hybrid As_2_S_3_/PMMA PCF for the transmission band edge A (black) and B (red).

The most important property of chalcogenide glasses is perhaps their ultra-high Kerr nonlinear coefficient, *n_2_*, which is 100 ~ 1000 times greater than fused silica and polymer^40^,^41^ and gives chalcogenide a very strongly intensity-dependent refractive index change, *Δn* = *n*_2_*I*, where I is the optical intensity. For the chalcogenide glass As_2_S_3_ we consider n_2_ = 2 × 10^−17^ m^2^/W at 800 nm^42^. If the chalcogenide films have a significant overlap with the guided mode, it can therefore be expected that the hybrid polymer PCF is nonlinear with transmission properties that can be tuned by changing the intensity. It has been demonstrated that the Kerr effect can provide an efficient way to control ultrafast optical component with intensity^43^,^44^,^45^. However, it should be noticed that the photosensitivity of chalcogenide glasses is an additional effect, which might also contribute to the change in resonance wavelength^46^. For that reason, further experiments using silica PCFs were performed in order to verify that the observed shift of the bands is due to the Kerr effect of the high index chalcogenide glass (see supplementary Figs. S5 and S6). Nonlinear effects have so far never been demonstrated in polymer PCFs, so the new hybrid polymer PCF could for the first time provide a pathway towards all-optical signal processing devices based on polymer PCFs. However, increasing the power may introduce thermal effects, which might also affect the guiding properties of the polymer PCF and compete with the intensity-induced changes. To discriminate between these two effects (thermal and nonlinear), we first investigated the response of the hybrid polymer PCF at different temperatures. Figure 5(a) shows the transmission at temperatures 23°C, 30°C, and 40°C, of the hybrid polymer PCF with 400 nm thick chalcogenide films in the holes. The spectrum of Fig. 5(a) at temperature 23°C (black curve) slightly differs from the one in Fig. 4(d) (red curve) since the spectrum is very sensitive to launching conditions. For this fiber the geometrically averaged refractive index of the holes is 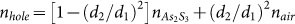, where *d*_1_ = 1.85 *μm* is the original air-hole diameter and *d*_2_ = 1.05 *μm* is the reduced diameter or the air hole after deposition of the 400 nm thick chalcogenide film. For the chalcogenide As_2_S_3_ we consider 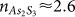 at these wavelengths^47^, which gives *n_hole_* ≈ 2.08 which is higher than the index of PMMA, *n_PMMA_* ≈ 1.5. Since the negative thermo-optic coefficient of the chalcogenide glass at short wavelengths^48^ is larger than the negative thermo-optic coefficient of PMMA (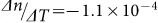 at 633 nm)^49^, the temperature-induced change in refractive index of chalcogenide glass (e.g. 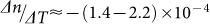 for As_2_S_3_ at 810 nm^50^) is dominating over the index change of PMMA. An increase in temperature will therefore decrease the refractive index contrast between the host polymer material and both the chalcogenide glass films and the average hole index. This change in the refractive index contrast directly influences the transmission bands by blue-shifting their location ~50 nm at 800 nm when the temperature increased from 23°C to 40°C. A similar blue-shift of the transmission bands has previously been reported in an ARROW guiding liquid-crystal filled polymer PCF^51^.

The characterization of the intensity-dependence (Kerr effect) of the hybrid polymer PCF was performed by placing a linearly variable optical attenuator between the active fiber and the source in order to control the power coupled into the fiber (see Methods). Figure 5(b) demonstrates the normalized transmission spectra of the hybrid polymer PCF for different power levels. The transmission band edges are consistently and linearly changing as the power is varying. By tracking the two main band edges at 610 nm (point A) and 825 nm (point B), a linear red-shift is observed as the power is increasing, as seen in Fig 5(c). This red-shift is due to the Kerr nonlinearity of the integrated chalcogenide As_2_S_3_ glass films inside the holes of the polymer PCF, which increases the index difference between the polymer and the chalcogenide glass when the power is increased^52^. The maximum shift of the first band edge (black line A) was found to be 6.1 nm for a maximum power change of 11.5 dB while for the longer wavelength edge (red line B) the shift was 17 nm since the chalcogenide-light interaction at longer wavelengths is higher. We would like to note that the starting wavelength in our experiments is ~500 nm, which is below the bandgap of the chalcogenide material and therefore the photosensitivity of the chalcogenide glass might could also have contributed to the red-shifting of the transmission band edges^46^. The numerical calculations have shown that (see supplementary Fig. S3) the estimated intensity required, in order to introduce a shift of 17 nm in the spectrum as shown in Fig. 5(c), is I = 7.4 × 10^14^ W/m^2^. However, the broadband supercontinuum source has not only a complicated spectrum, it also has a complicated temporal profile, consisting of a sea of small pulses around the original pump (here 1064 nm) and many high-amplitude pulses in the near-infrared part, group-velocity matched to so-called low-amplitude dispersive wave packets in the blue part of the spectrum (see supplementary Fig. S4). This makes it difficult to separate the influence of the different spectral parts of the supercontinuum on the observed nonlinear effect in the hybrid polymer PCF. In addition the spatial mode-profile at wavelengths close to the band edge is highly complicated with a lot of light being present in the chalcogenide layers. Combined this means it is most relevant to look at the local intensity required in the films in order to observe the nonlinear shift in the band edge.

## Discussion

In summary, we have demonstrated the first PMMA-based polymer PCF integrated with highly nonlinear chalcogenide As_2_S_3_ nanofilms. The deposition of the chalcogenide films inside the holes of the polymer PCF was achieved at room temperature with a glass solution-based infiltration method followed by a drying process to evaporate the solvent and leave chalcogenide glass nanofilms with a controlled thickness determined by the concentration. The transmission properties of the fiber display clear antiresonance guiding phenomena and the strong Kerr nonlinearity of the chalcogenide glass films allows the possibility to shift the resonance edges as much as 17 nm around 825 nm. The proposed integration method allows the deposition of nanofilms inside the holes of the polymer PCF using an unlimited number of different nonlinear glasses and even allows multi-layer deposition of different glasses. The unique advantages of chalcogenide glasses and the polymer PCF platform, combined with the proposed fabrication method, opens the way towards novel all-fiber nonlinear devices. However, the proposed hybrid structure has high loss compared to single-material silica or polymer fibers. These losses are mainly introduced by the polymer and chalcogenide material absorptions, scattering from the glass-polymer interfaces in the holes as well as imperfections of the structure during the fabrication of the fiber. Therefore, further research is required in order to reduce these losses. Possible ways to improve the performance of the hybrid fiber include using other polymer materials and improve the control of the growth and deposition of the chalcogenide glass films.

## Methods

### Fiber fabrication

The polymer PCF used in our experiments was fabricated in-house on a custom-built polymer PCF draw tower using a two-stage (cane-sleaving) drawing process of a drilled preform^53^. A primary PMMA cylindrical preform of diameter 6 cm and length 10 cm was drilled with the desired 3-ring hole pattern using a computer controlled (CNC) drilling machine. The preform was first drawn to a cane of 5 mm diameter. The cane was sleeved with two polymer tubes and this secondary preform was then drawn to ~125 μm in diameter fiber, giving several 100 m of fiber. The structural dimensions of the fiber can be controlled by adjusting the drawing parameters, such as the temperature, the pressure applied to the holes, the preform feed rate, and the fiber draw speed.

### Nanocolloidal solution-based chalcogenide glass preparation

Bulk As_2_S_3_ is typically made using the melt-quenching technique, where the required chemical amounts of high purity As and S are mixed in a sealed quartz ampoule and heated to ~700°C for 48 h in a rocking furnace, before it is quenched to room temperature^28^. However, in this work commercially available high purity (99.999%) As_2_S_3_ bulk pieces were used (purchased from Alfa Aesar) avoiding thus the first preparation step. The bulk As_2_S_3_ glass was grinded into fine powder using a ceramic mortar in an N_2_ environment and then dissolved in n-butylamine (purity > 99%) inside a sealed glass container to prevent any solvent evaporation. A hot plate with magnetic stirrer was used to expedite the dissolution process. The whole procedure took several days in order to ensure complete homogenization and dissolution of the bulk glass.

### Optical characterization

**Figure 6:**
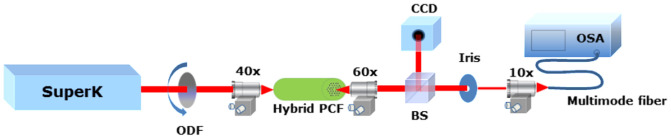
Experimental set up used for the optical characterization of the hybrid PCF.

The optical characterization of the hybrid polymer PCF was made using a high power supercontinuum laser source (SuperK Versa, NKT Photonics A/S) with average power ~ 1.5 W over the 480–2200 nm wavelength region. The light was coupled into the hybrid polymer PCF using a 40× microscope objective. The output beam was collimated with another 60× microscope objective and focused into a multimode fiber (60 μm core size) and the signal was finally recorded with an optical spectrum analyzer - OSA (Ando AQ6317B) with minimum resolution 0.05 nm. All the undesired light was blocked by using an iris at the output, such that only light from the core was detected. A beam splitter (BS) was also inserted between the output and the optical spectrum analyzer to capture the near-field profile of the fundamental core mode and the high-index cladding modes with a high resolution CCD camera. For the temperature measurements presented in Figure 5(a) in the manuscript, a controlled heating element (Linkam MC60) was placed in contact with the fiber. For the intensity measurements presented in Figure 5 (b), a linear optical density filter (ODF) was inserted between the source and the fiber to control the input power. The experimental set up is shown in Fig. 6. For the intensity measurements, we ensured that the temperature and the fiber were at equilibrium, by recording the signal 10 minutes after the change of the power, in order to leave sufficient time for the fiber temperature to have reached a stationary value. The total duration of the experiment was ~40 minutes. The same time intervals applied for the temperature measurements as well.

## Supplementary Material

Supplementary Information Hybrid polymer photonic crystal fiber with integrated chalcogenide glass nanofilms

## References

[b1] Birks T. A., Knight J. C. & Russell P. St. J. Endlessly single-mode photonic crystal fiber. Opt. Lett. 22, 961–963 (1997). 18185719 10.1364/ol.22.000961

[b2] Ranka J. K., Windeler R. S. & Stentz A. J. Visible continuum generation in air-silica microstructure optical fibers with anomalous dispersion at 800 nm. Opt. Lett. 25, 25 (2000). 18059770 10.1364/ol.25.000025

[b3] Cregan R. F. *et al.* Single-mode photonic band gap guidance of light in air. Science 285, 1537 (1999). 10477511 10.1126/science.285.5433.1537

[b4] Monro T. M., West Y. D., Hewak D. W., Broderick N. G. R. & Richardson D. J. Chalcogenide holey fibres. Electron. Lett. 36, 1998 (2000).

[b5] Brilland L. *et al.* A. Fabrication of complex structures of Holey Fibers in Chalcogenide glass. Opt. Express 14, 1280 (2006). 19503451 10.1364/oe.14.001280

[b6] Eijkelenborg M. *et al.* Microstructured polymer optical fibre. Opt. Express 9, 319 (2001). 19421303 10.1364/oe.9.000319

[b7] Emiliyanov G. *et al.* Localized biosensing with Topas microstructured polymer optical fiber. Opt. Lett. 32, 460 (2007). 17392887 10.1364/ol.32.000460

[b8] Emiliyanov G. *et al.* Localized biosensing with Topas microstructured polymer optical fiber: erratum. Opt. Lett. 32, 1059 (2007). 10.1364/ol.32.000460 17392887

[b9] Markos C. *et al.* High-Tg TOPAS microstructured polymer optical fiber for fiber Bragg grating strain sensing at 110 degrees. Opt. Express 21, 4758 (2013). 23482009 10.1364/OE.21.004758

[b10] Fink Y. *et al.* A Dielectric Omnidirectional Reflector. Science 282, 1679 (1998). 9831553 10.1126/science.282.5394.1679

[b11] Bayindir M. *et al.* Metal–insulator–semiconductor optoelectronic fibres. Nature 431, 826 (2004). 15483607 10.1038/nature02937

[b12] Abouraddy A. F. *et al.* Towards multimaterial multifunctional fibres that see, hear, sense and communicate. Nat. Mater. 6, 336 (2007). 17471274 10.1038/nmat1889

[b13] Dudley J. M. & Taylor J. R. Ten years of nonlinear optics in photonic crystal fibre. Nat. Photonics 3, 85 (2009).

[b14] Johnson I. P., Kalli K. & Webb D. J. 827 nm Bragg grating sensor in multimode microstructured polymer optical fiber. Electron. Lett. 46, 1217 (2010).

[b15] Large M. C. J., Moran J. & Ye L. The role of viscoelastic properties in strain testing using microstructured polymer optical fibres (mPOF). Meas. Sci. Technol. 20, 034014 (2009).

[b16] Johnson I. P. *et al.* Optical fibre Bragg grating recorded in TOPAS cyclic olefin copolymer. Electron. Lett. 47, 271 (2011).

[b17] Yuan W. *et al.* Humidity insensitive TOPAS polymer fiber Bragg grating sensor. Opt. Express 19, 19731 (2011). 21996915 10.1364/OE.19.019731

[b18] Jensen J. *et al.* Selective detection of antibodies in microstructured polymer optical fibers. Opt. Express 13, 5883 (2005). 19498594 10.1364/opex.13.005883

[b19] Emiliyanov G., Høiby P. E., Pedersen L. H. & Bang O. Selective Serial Multi-Antibody Biosensing with TOPAS Microstructured Polymer Optical Fibers. Sensors 13, 3242 (2013). 23529122 10.3390/s130303242 PMC3658743

[b20] Litchinitser N. M., Abeeluck A. K., Headley C. & Eggleton B. J. Antiresonant reflecting photonic crystal optical waveguides. Opt. Lett. 27, 1592 (2002). 18026511 10.1364/ol.27.001592

[b21] Luan F. *et al.* All-solid photonic bandgap fiber. Opt. Lett. 20, 2369 (2004). 10.1364/ol.29.002369 15532270

[b22] Larsen T., Bjarklev A., Hermann D. & Broeng J. Optical devices based on liquid crystal photonic bandgap fibres. Opt. Express 11, 2589 (2003). 19471372 10.1364/oe.11.002589

[b23] Kerbage C. *et al.* Highly tunable birefringent microstructured optical fiber. Opt. Lett. 27, 842 (2002). 18007946 10.1364/ol.27.000842

[b24] Schmidt M. A. *et al.* All-solid bandgap guiding in tellurite-filled silica photonic crystal fibers. Opt. Lett. 34, 1946 (2009). 19571961 10.1364/ol.34.001946

[b25] Konidakis I., Zito G. & Pissadakis S. Photosensitive, all-glass AgPO3/silicaphotonic bandgap fiber. Opt. Lett. 37, 2499 (2012). 22743434 10.1364/OL.37.002499

[b26] Grujic T., Kuhlmey B. T., Argyros A., Coen S. & Martijn de Sterke C. Solid-core fiber with ultra-wide bandwidth transmission window due to inhibited coupling. Opt. Express 18, 25556 (2010). 21164901 10.1364/OE.18.025556

[b27] He R. *et al.* Integration of gigahertz-bandwidth semiconductor devices inside microstructured optical fibres. Nat. Photonics 6, 174 (2012).

[b28] Markos C., Yannopoulos S. N. & Vlachos K. Chalcogenide glass layers in silica photonic crystal fiber. Opt. Express 20, 14814 (2012). 22772176 10.1364/OE.20.014814

[b29] Kuhlmey B. T., Eggleton B. J. & Wu D. K. C. Fluid-Filled Solid-Core Photonic Bandgap Fibers. J. Lightwave Technol. 27, 1617 (2009).

[b30] Eggleton B. J., Luther-Davies B. & Richardson K. Chalcogenide photonics. Nat. Photonics 5, 141 (2011).

[b31] Chern G. C. & Lauks I. Spin-coated amorphous-chalcogenide films. J. Appl. Phys. 53, 6979 (1982).

[b32] Da N., Wondraczek L., Schmidt M. A., Granzow N. & Russell P. St. J. High index-contrast all-solid photonic crystal fibers by pressure-assisted melt infiltration of silica matrices. J. Non-Cryst. Solids 356, 1829 (2010).

[b33] Tsay C., Mujagić E., Madsen C. K., Gmachl C. F. & Arnold C. B. Mid-infrared characterization of solution-processed As_2_S_3_ chalcogenide glass waveguides. Opt. Express 18, 15523 (2010). 20720932 10.1364/OE.18.015523

[b34] Kuhlmey B. T., McPhedran R. C. & Martijn de Sterke C. Modal cutoff in microstructured optical fibers. Opt. Lett. 27, 1684 (2002). 18033335 10.1364/ol.27.001684

[b35] Guiton T. A. & Pantano C. G. Solution/gelation of arsenic trisulfide in amine solvents. Chem. Mater. 1, 558 (1989).

[b36] Stefani A., Nielsen K., Rasmussen H. K. & Bang O. Cleaving of TOPAS and PMMA microstructured polymer optical fibers: Core-shift and statistical quality optimization. Opt. Commun. 285, 1825 (2012).

[b37] Tyagi H. K., Schmidt M. A., Prill Sempere L. & Russell P. S. Optical properties of photonic crystal fiber with integral micron-sized Ge wire. Opt. Express 16, 17227–17236 (2008). 18958003 10.1364/oe.16.017227

[b38] Litchinitser N. *et al.* Application of an ARROW model for designing tunable photonic devices. Opt. Express 12, 1540 (2004). 19474979 10.1364/opex.12.001540

[b39] Kuhlmey B. T., Pathmanandavel K. & McPhedran R. C. Multipole analysis of photonic crystal fibers with coated inclusions. Opt. Express 14, 10851–10864 (2006). 19529498 10.1364/oe.14.010851

[b40] Slusher R. E. *et al.* Raman gain and nonlinear phase shifts in high-purity As_2_Se_3_ chalcogenide fibers. J. Opt. Soc. Am. B 21, 1146 (2004).

[b41] Baker C. & Rochette M. Highly nonlinear hybrid AsSe-PMMA microtapers. Opt. Express 18, 12391 (2010). 20588365 10.1364/OE.18.012391

[b42] Zakery A., Ruan Y., Rode A. V., Samoc M. & Luther-Davies B. Low-loss waveguides in ultrafast laser-deposited As_2_S_3_ chalcogenide films. J. Opt. Soc. Am. B 20, 1844–1852 (2003).

[b43] Lenz G. *et al.* Large Kerr effect in bulk Se-based chalcogenide glasses. Opt. Lett. 25, 254–256 (2000). 18059846 10.1364/ol.25.000254

[b44] Vukovic N. *et al.* Ultrafast optical control using the Kerr nonlinearity in hydrogenated amorphous silicon microcylindrical resonators. Sci. Rep. 3, 2885 (2013). 24097126 10.1038/srep02885 PMC3791441

[b45] Qin F., Meng Z.-M., Zhong X.-L., Liu Y. & Li Z. Y. Fabrication of semiconductor-polymer compound nonlinear photonic crystal slab with highly uniform infiltration based on nano-imprint lithography technique. Opt. Express 20, 13091–13099 (2012). 22714336 10.1364/OE.20.013091

[b46] Benoit G., Kuriki K., Viens J.-F., Joannopoulos J. D. & Fink Y. Dynamic all-optical tuning of transverse resonant cavity modes in photonic bandgap fibers. Opt. Lett. 30, 1620 (2005). 16075516 10.1364/ol.30.001620

[b47] Rodney W. S., Malitson I. H. & King T. A. Refractive index of arsenic trisulfide. J. Opt. Soc. Am. 48, 633 (1958).

[b48] Yaman M., Kondakci H. E. & Bayindir M. Large and dynamical tuning of a chalcogenide Fabry-Perot cavity mode by temperature modulation. Opt. Express 18, 3168 (2010). 20174155 10.1364/OE.18.003168

[b49] Kang E. S., Bae J. U. & Bae B. S. Measurement of Thermo-Optic Coefficients in Sol-Gel Hybrid Glass Films. J. Sol–Gel Sci. Tech. 26, 981 (2003).

[b50] Zakery A. Low loss waveguides in pulsed laser deposited arsenic sulfide chacogenide films. J. Phys. D 35, 2909 (2002).

[b51] Yuan W., Wei L., Alkeskjold T. T., Bjarklev A. & Bang O. Thermal tunability of photonic bandgaps in liquid crystal infiltrated microstructured polymer optical fibers. Opt. Express 17, 19356 (2009). 19997156 10.1364/OE.17.019356

[b52] Tonglei C. *et al.* Numerical Simulation of Dynamic Bandgap Control in All-Solid Chalcogenide–Tellurite Photonic Bandgap Fiber. IEEE Phot. J. 5, 2202206 (2013).

[b53] Barton G., Eijkelenborg M. A., Henry G. J., Large M. C. & Zagari J. Fabrication of microstructured polymer optical fibres. Opt. Fiber Technol. 10, 325 (2004).

