# Supplementary material for: Hybrid polymer photonic crystal fiber with integrated chalcogenide glass nanofilms
Source: Sci Rep. 2014 Aug 14;4:6057. doi: 10.1038/srep06057 (PMC5377533; doi:10.1038/srep06057)
Supplement: Supplementary Information — Hybrid polymer photonic crystal fiber with integrated chalcogenide glass nanofilms [file srep06057-s1.pdf]

Supplementary Information:

Hybrid polymer photonic crystal fiber with integrated chalcogenide glass nanofilms

Christos Markos,* Irnis Kubat , and Ole Bang

DTU Fotonik, Department of Photonics Engineering, Technical University of Denmark, DK-2800 Kgs. Lyngby, Denmark

Scanning Electron Microscopy

After the deposition of the chalcogenide glass inside the holes of the polymer PCF, the end facet was cleaved using the hot-blade/fiber technique[1] to obtain a good quality smooth surface for the SEM. For 125 μm diameter polymer PCFs of PMMA, it has been shown that the optimum cleaving temperature is 77.5°C for both the fiber and the cleaving blade[1]. The SEM images were taken with an FEI Quanta 200 ESEM FEG Electron Microscope using an accelerating voltage of 2-10kV. The polymeric nature of the fiber sets a limitation on the applied acceleration voltage and consequently it was not possible to achieve high magnification levels in order to determine the exact thickness of the deposited chalcogenide glass films of a few nanometers.

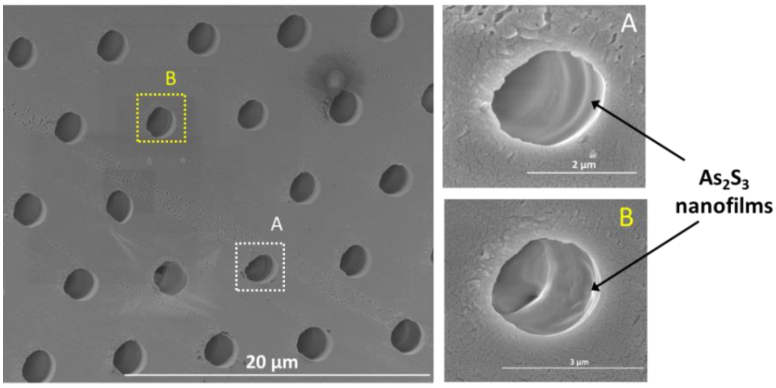

Figure S1: Scanning Electron Microscope image of the hybrid polymer PCF (50mg/ml concentration) cleaved after ~2 cm. The two SEM images (A and B) indicate clearly the thin nanometer-scaled films at the inner surface of the holes.

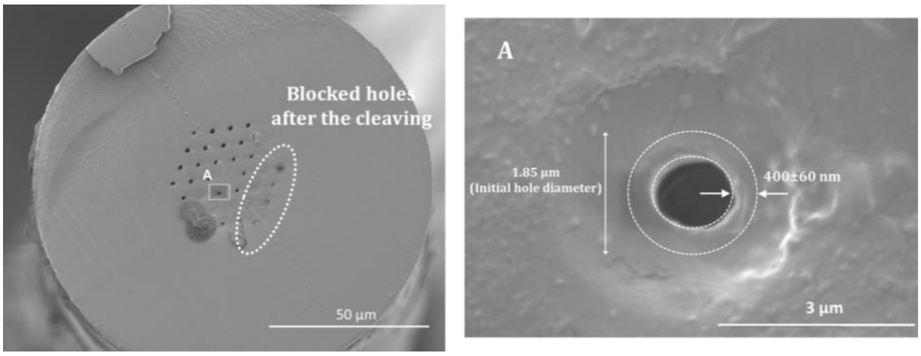

Figure S2: Scanning Electron Microscope image of the hybrid polymer PCF (400mg/ml) cleaved after 2 cm. The SEM image (A) indicates clearly the ~400nm thick film at the inner surface of the holes.

EDX analysis

The authors applied energy dispersive X-ray Spectroscopy (EDX) (using an Oxford Instruments 80 mm^2 X-Max silicon drift detector) in order to confirm the existence of the two main elements of the chalcogenide nanofilms Arsenic (As) and Sulfide (S). For very thin films, the accuracy of the EDX spectroscopy technique is sometimes limited because it depends on the nature of the target material. In this work, however, the dense nature of the chalcogenide glass allowed us to determine the elements (As and S) of the formed films inside the holes of the polymer PCF. The acceleration voltage was 20kV while the relatively long acquisition time allowed us to observe the weak As and S peaks in the EDX spectrum.

Simulations

The numerical calculations of the guiding properties of the hybrid fiber (Fig. 4(a)) were performed using a Finite Element Method (COMSOL Multiphysics v.4.3b). The core area of the polymer photonic crystal fiber (PCF) was defined as $2\Lambda - \mathrm{d}$ where Λ is the distance between the holes and d is the air-hole diameter. The fundamental guided mode was defined as the core mode with the highest fraction of power in the core. The dispersion of As_2S_3 and PMMA were both included in our calculations based on their Sellmeier equations but both materials were considered lossless. The Sellmeier equation for the chalcogenide glass As_2S_3 is described by[2]:

$$n(\lambda) = \sqrt{1 + \sum_{i=1}^{5} \frac{A_i * \lambda^2}{\lambda^2 - \lambda_i^2}}$$

The Sellmeier coefficients of As_2S_3 are shown in the following Table 1:

i	1	2	3	4	5
A_i	1.898368	1.922298	0.8765138	0.118878	0.956998
$\lambda_i[\mu m]$	0.1500	0.250	0.350	0.450	27.3861

Table S1. Sellmeier coefficients of Arsenic Trisulfide (As_2S_3)[2]

Similarly, the Sellmeier equation for PMMA is given by[3]:

$$n\,(\lambda) = \sqrt{1 + \sum_{i=1}^{3} \frac{A_i * \lambda^2}{\lambda^2 - \lambda_i^2}}$$

The Sellmeier coefficients of PMMA can be found in the following Table 2.

i	1	2	3
A_i	0.4963	0.6965	0.3223
$\lambda_i[\mu m]$	71.8000	117.4000	9237

Table S2. Sellmeier coefficients of PMMA[3]

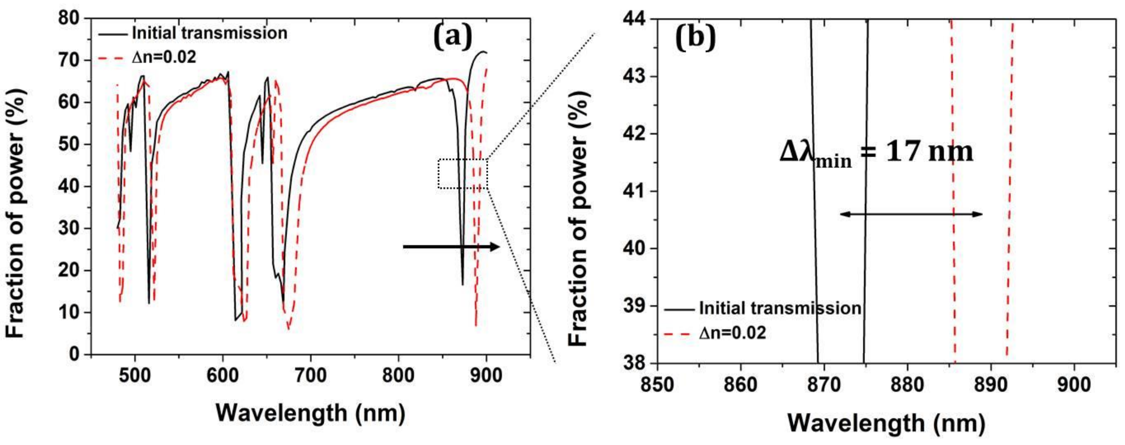

Figure S3: (a) Numerical calculations of the transmission spectrum after a uniform change of the refractive index of the high index glass layer by 0.02 (b) Zoomed spectrum of the long band edge showing a 17nm shift.

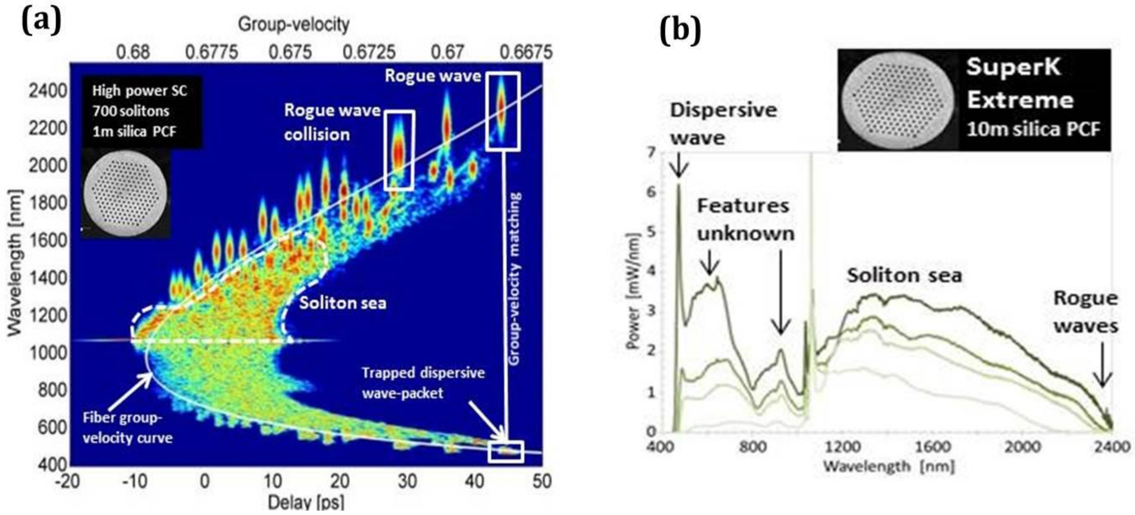

Figure S4: (a) Simulated spectrogram at the output of the supercontinuum source showing the generation of dispersive waves below zero-dispersion wavelength (ZDW) and solitons above the ZDW. (b) Actual output spectrum of a commercial supercontinuum source.

Experiments using silica PCF

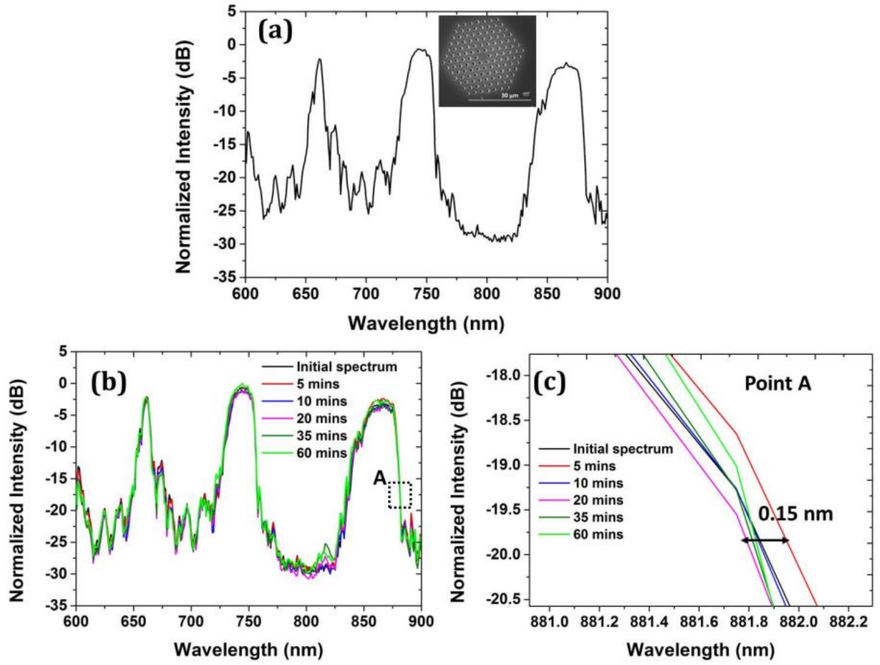

Figure S5: (a) Transmission spectrum of the hybrid silica PCF filled with As_2S_3 at maximum output power of the source (~1.5W). Inset: SEM image of the silica PCF used in the experiments. (b) Evolution of the transmission bands over time. (c) Zoomed long band edge (point A) showing only a 0.15nm fluctuation over 60 minutes.

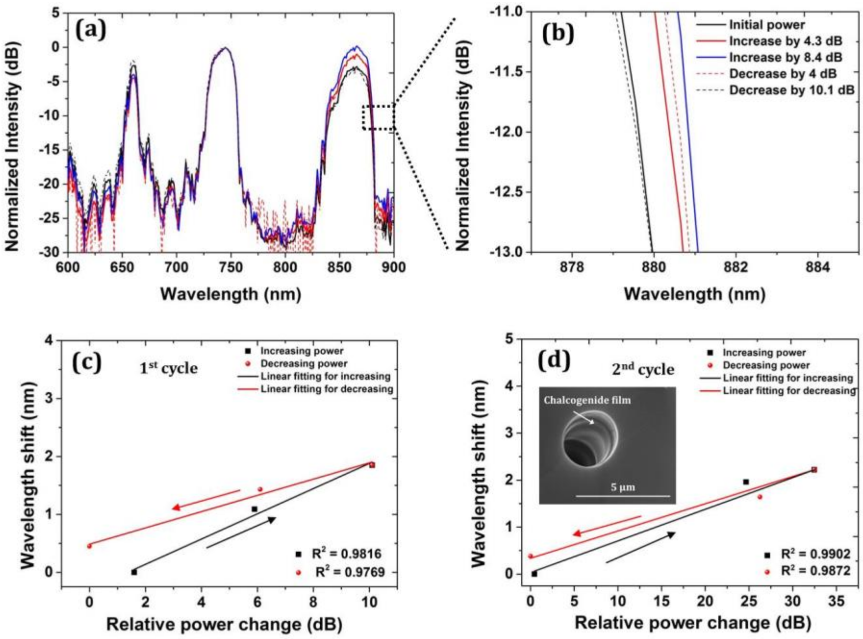

Figure S6: (a) Transmission spectrum of the hybrid silica PCF filled with As_2S_3 at different powers. (b) Long wavelength edge shift with total power. (c) 1st cycle of measurements showing the wavelength shift versus power (relative power change 0 – 10dB). (d) 2nd cycle of measurements indicating the repeatable wavelength shift of the long wavelength edge (relative power change 0-32.4dB). Inset: SEM image of a single hole coated with a few nanometers thickness chalcogenide glass.

References

1. Stefani, A., Nielsen, K., Rasmussen, H. K., Bang, O. Cleaving of TOPAS and PMMA microstructured polymer optical fibers: Core-shift and statistical quality optimization. *Opt. Commun.* **285**, 1825 (2012).

2. Rodney, W. S., Malitson, I. H., King, T.A. Refractive index of arsenic trisulfide. *J. Opt. Soc. Am.* **48**, 633 (1958).

3. Ishigure, T., Nihei, E., Koike, Y., Optimum refractive-index profile of the graded-index polymer optical fiber, toward gigabit data links. *Appl. Opt.* **35,** 2048 (1996).
